# Tanzanian women´s knowledge about Cervical Cancer and HPV and their prevalence of positive VIA cervical screening results. Data from a Prevention and Awareness Campaign in Northern Tanzania, 2017 – 2019

**DOI:** 10.1080/16549716.2020.1852780

**Published:** 2020-12-28

**Authors:** Antje Henke, Ulrike Kluge, Theda Borde, Bariki Mchome, Furaha Serventi, Oliver Henke

**Affiliations:** a Kilimanjaro Christian Medical Centre, Cancer Care Centre, Moshi, Tanzania; b Department of Psychiatry and Psychotherapy, Charité Universitätsmedizin, Berlin, Germany; c Alice Salomon Hochschule Berlin, University of Applied Sciences, Berlin, Germany; d Department of Gynaecology, Kilimanjaro Christian Medical Centre, Moshi, Tanzania

**Keywords:** Cervical Cancer, cancer prevention, via screening, HPV, Tanzania

## Abstract

**Background**: 14.9 million women (≥15 years) in Tanzania are at risk of developing cervical cancer.

Limited cancer care facilities, prevention programs and sparse knowledge among community members and healthcare workers contribute to late-stage presentation leading to a high mortality rate.

**Objective**: This study aims to scientifically accompany prevention and awareness campaigns (PrevACamp) in northern Tanzania in its real-world settings to obtain (1) a better understanding about cervical cancer and HPV knowledge amongst female PrevACamp participants and (2) to determine the prevalence of pre-cancerous lesions among women undergoing cervical cancer VIA screening.

**Method**: Cross-sectional survey among PrevACamp attendees in two regions in Northern Tanzania. Two data collections tools were used: Questionnaires and clinical data from VIA screening. Data were collected from October 2017 to March 2019.

**Results**: 2,192 PrevACamp attendees were interviewed and 2,224 received VIA screening. There was significant nescience on cervical cancer regardless of education level, resident status, or number of children as well as nescience on HPV in all age groups, especially in urban areas and misconceptions about cancer. Screening revealed VIA positivity rate of 3.1%.

**Conclusion**: There is an alarming lack of knowledge about cervical cancer and, to a lesser Extent, about HPV among the study participants. Having health insurance influenced the level of knowledge significantly. Outreach programs in rural areas appear to target the population in need of health education. Low positive VIA screening results are paralleled with lower HIV rates among the women. We assume that the high density of primary health care coverage in northern Tanzania contributes to these findings..

## Background

Cervical cancer is the fourth most common cause of cancer-related deaths with more than 300,000 cases per year worldwide [1,2]. Approximately 90% of all cervical cancer deaths occur in low- and middle-income countries (LMICs) [2]. In Sub-Saharan Africa (SSA), it is the second leading cause of cancer-related deaths among women [3] and in Tanzania, cervical cancer is the most frequently diagnosed cancer among women aged between 15 and 44 years [4]. The annual incidence of cervical cancer is 9,770 cases per 100,000 women with a mortality rate of 6,695 [4]. If no specific action is taken, Tanzania is estimated to have 12,416 new cervical cancer cases and 9,923 deaths per year in 2025 [5].

Comprehensive national screening programs may reduce the incidence and mortality rate from cervical cancer [6]. Limited access to these programs in LMICs increases the prevalence of advanced stages of the disease. This compares to high-income countries where primary and secondary prevention programs lead to early detection and increased survival rates [2,6]. Currently, the national cervical cancer screening programme in Tanzania uses VIA (visual inspection with acetic acid) as the standard screening procedure [7] which is available free of charge in government and church-based hospitals on different levels of care. Although, VIA has lower sensitivity and specificity compared to PAP smear and HPV testing [8], it remains the standard of care in many low income countries because of its single visit approach and the generally high prevalence of cervical cancer in these countries. PAP smears and HPV (human papillomavirus) tests are available in the zonal hospitals in Tanzania.

Human papillomavirus (HPV) type 16 and 18 cause 70% of cervical cancer and pre-cancerous cervical lesion cases [9]. The World Health Organization (WHO) recommends the following measures to lower the burden of cervical cancer: (1) primary prevention (HPV vaccination for girls aged 9–14 years, so they are protected before they become sexually active), (2) secondary prevention (screening and treatment of pre-cancerous lesions), (3) tertiary prevention (diagnosis and treatment of invasive cervical cancer) and (4) palliative care [2].

In general, infection-related cancer occurs more often in SSA compared to other regions in the world [10]. Apart from HPV infections, women with human immunodeficiency virus (HIV) have a higher likelihood of developing cervical cancer [11]. The HIV-prevalence of women above 15 years of age in Tanzania is 4.6% [12] and affecting urban and rural areas alike. Nearly 80% are resulting from heterosexual transmission [13].

The 14.9 million women that are above the age of 15 years in Tanzania are at risk of developing cervical cancer [4]. Kilimanjaro Christian Medical Center (KCMC) hosts the only specialized cancer care facility in Northern Tanzania [14] with a catchment area of approximately 15 million people. The next available radiation unit is located 550 km away in Dar es Salaam, Tanzania´s largest city. The Tanzanian Ministry of Health and Social Welfare (MoHSW) implemented a National Cancer Control Strategy (NCCS), that targets cancer education in schools, HPV vaccination, health promotion and screening programs for high-risk populations [15].

In 2014, a schools-based HPV vaccination program supported by the GAVI-Alliance (Global Alliance for Vaccines and Immunization) was successfully piloted within the Kilimanjaro Region [16,17]. An increase in national vaccination programs for girls between 9 and 14 years is expected in the future [16], which will need support from prevention and awareness campaigns.

A lack of knowledge about preventive measures among the general population and healthcare workers hinders effective cervical cancer prevention and treatment [18,19] and must be considered when designing prevention strategies. It is known that knowledge gaps among health care workers and the general population lead to a higher mortality rate in LMICs [19,20]. Furthermore, differences in knowledge have been found between rural and urban areas in Tanzania [21,22], with generally lower knowledge in remote areas. However, evidence regarding cervical cancer and HPV knowledge as well as the prevalence of precancerous cervical lesions are sparse.

In 2017, the Cancer Care Centre (CCC) at KCMC launched Cancer Prevention and Awareness Campaigns (PrevACamps) in two Northern Tanzanian regions (Kilimanjaro and Arusha) covering the districts of Hai, Mwanga, Rombo, Moshi Urban, Arusha Urban, Moshi Rural, and Siha. The PrevACamps offer education seminars and screening programs for the communities, as well as training community health care providers to enhance their cancer awareness and knowledge. This study aims to scientifically accompany PrevACamp in its real world setting to obtain (1) a better understanding about cervical cancer and HPV knowledge amongst female PrevACamp participants and (2) to determine the prevalence of pre-cancerous lesions among the screened women. The study focuses on the differences between women living in rural and urban settings.

## Methods

**Figure 1.**
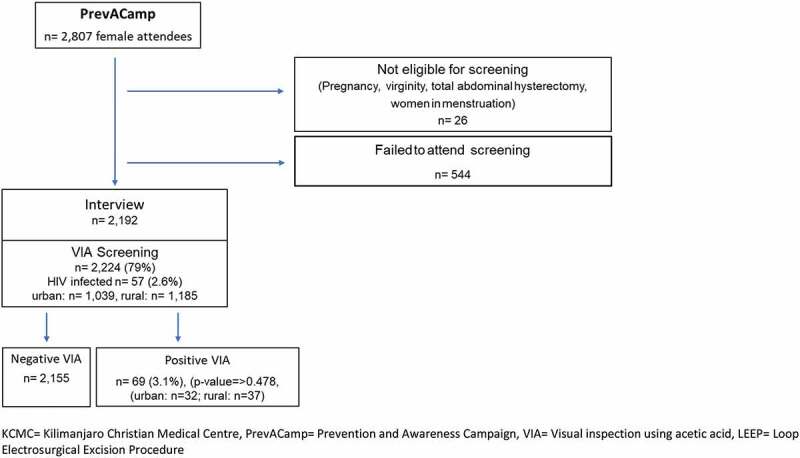
Flowchart of attendee´s recruitment

A cross-sectional study design among PrevACamp attendees was chosen. Two data collection tools were used: questionnaires and documented clinical data records from the mass screening during PrevACamps between October 2017 and March 2019. Cervical cancer screening was conducted by applying VIA [23] followed by cryotherapy where indicated. (Figure 1)

KCMC = Kilimanjaro Christian Medical Centre, PrevACamp = Prevention and Awareness Campaign, VIA = Visual inspection using acetic acid, LEEP = Loop Electrosurgical Excision Procedure.

### Study setting

Arusha (with a population of 1,694,310) and Kilimanjaro (1,640,087) Regions (districts of Hai, Mwanga, Rombo, Moshi Urban, Arusha Urban, Moshi Rural, and Siha) are characterized by rural and urban areas. The majority of inhabitants live from small-scale farming or day labor jobs and small businesses [24]. The PrevACamp events were conducted in either faith-based hospitals or health centers in the respective districts. The regional and district medical officers were involved in planning and conducting the events.

### Study population and recruitment

The attendees were invited through loudspeaker cars, church announcements, and radio advertisements in the respective districts. Questionnaires: Interviewees were recruited from all PrevACamp attendees using convenience sampling of just arriving attendees. Trained interviewers informed all prospective interviewees about the purpose of the study and obtained consents.

VIA-screening: Cervical screening was offered to all female PrevACamp participants above the age of 18 years. Prior to screening, all women were informed by nurses about the screening process and possible results. Exclusion criteria were pregnancy, virginity, women with total abdominal hysterectomy (TAH), and women who were menstruating (Figure 1).

### Data collection tools

Data collection tools were questionnaires and clinical data from the VIA screenings.

Questionnaires: Questions from the validated Cervical Cancer Awareness Measure (Cervical CAM) were selected [25]. After discussions with key informants, questions were rephrased, and additional questions were added for cultural and social adaptation into the Tanzanian setting. The questionnaire was developed in English and for- and back-translated by two independent Swahili speakers to assure the coequality of the Swahili questionnaire. The questionnaire was divided into five sections: (1) cancer knowledge, (2) risk factors, (3) early symptoms, (4) cancer beliefs, and (5) socio- demographic characteristics. The survey included in total 62 items: 22 closed responses (yes/no/I do not know), 14 open-ended, 9 multiple response questions and 17 questions about socio-demographic characteristics. Four questions were asked about cervical cancer knowledge and three questions about HPV knowledge.

Average interview time was 25 minutes and the questionnaires were administered by 2 male and 3 female interview-trained health care professionals. 2 pilot phases were conducted for evaluation of feasibility and comprehension of the questions.(Figure 2Figure 2Figure 2Figure 2Figure 2)

**Figure 2.**
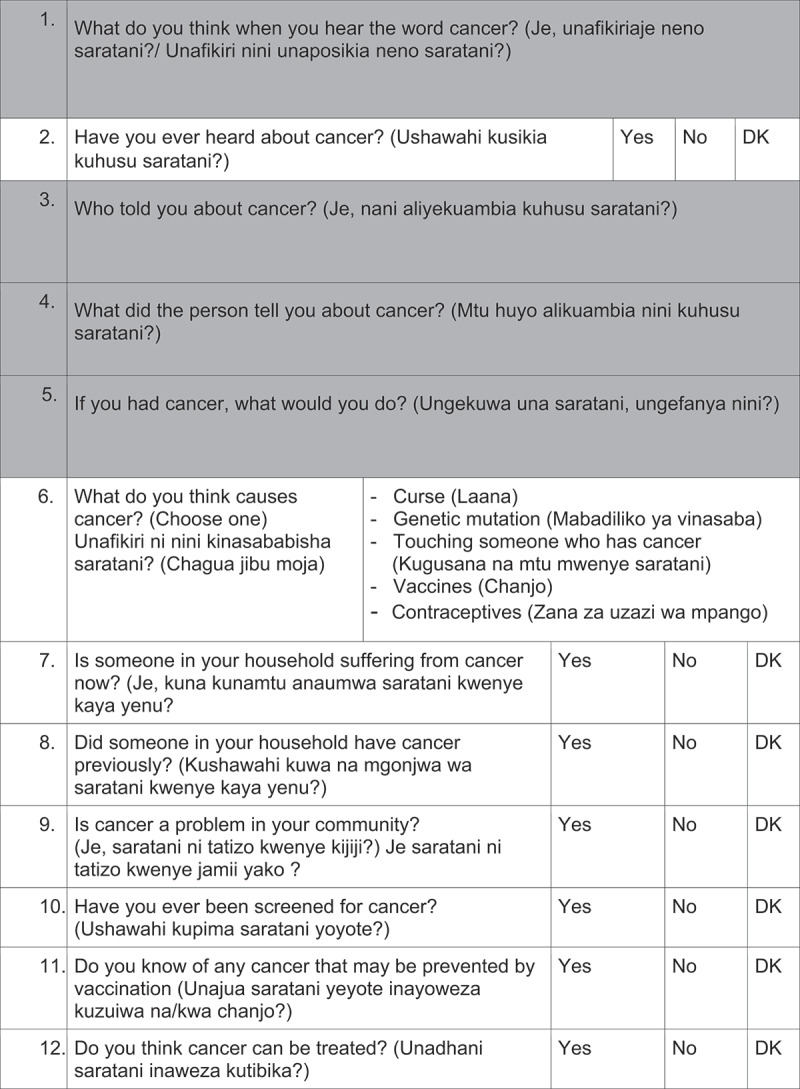
Questionnaire

DK: don´t know, The questions highlighted in grey were not analyzed in this study.

**Figure 2.**
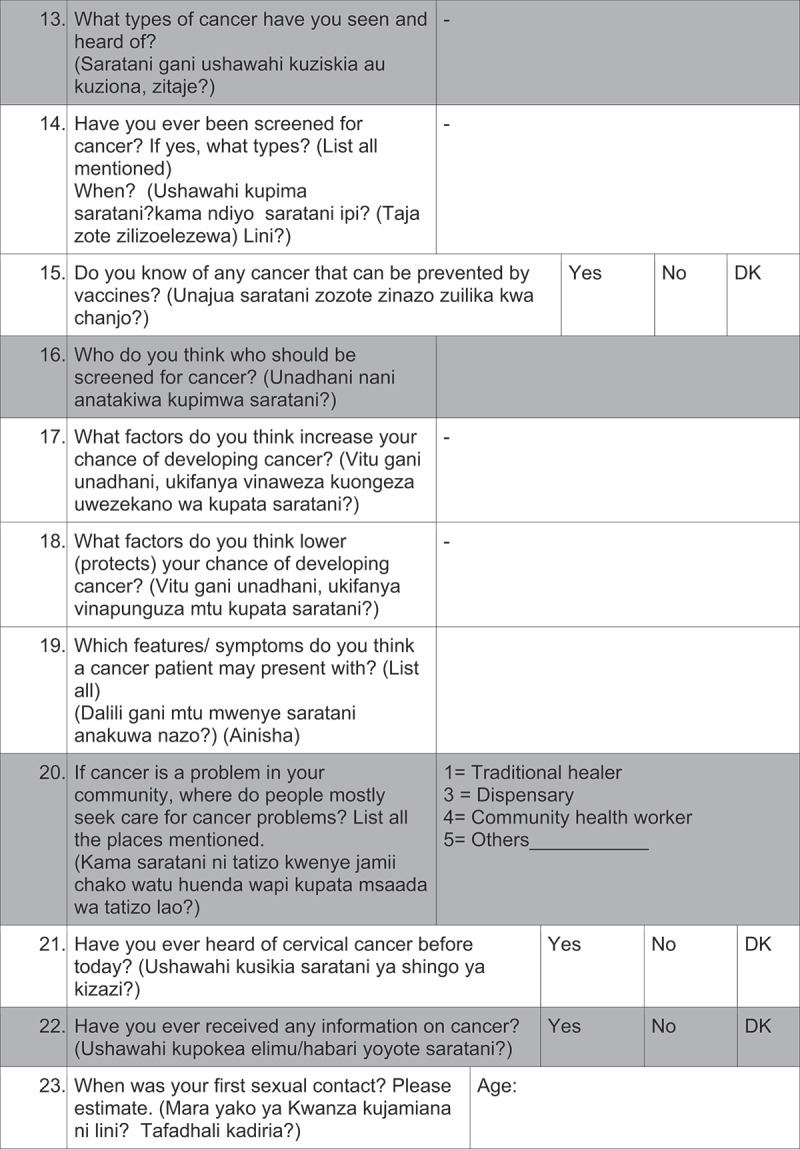
(Continued)

**Figure 2.**
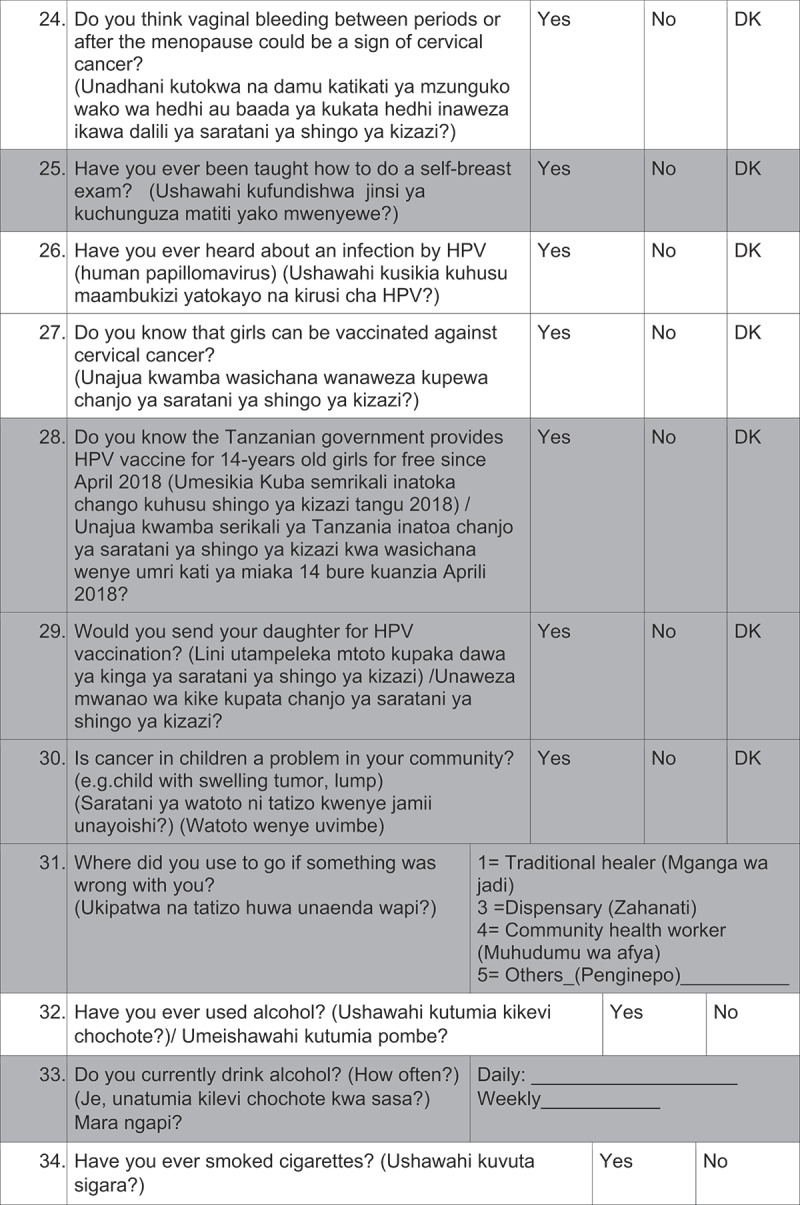
(Continued)

**Figure 2.**
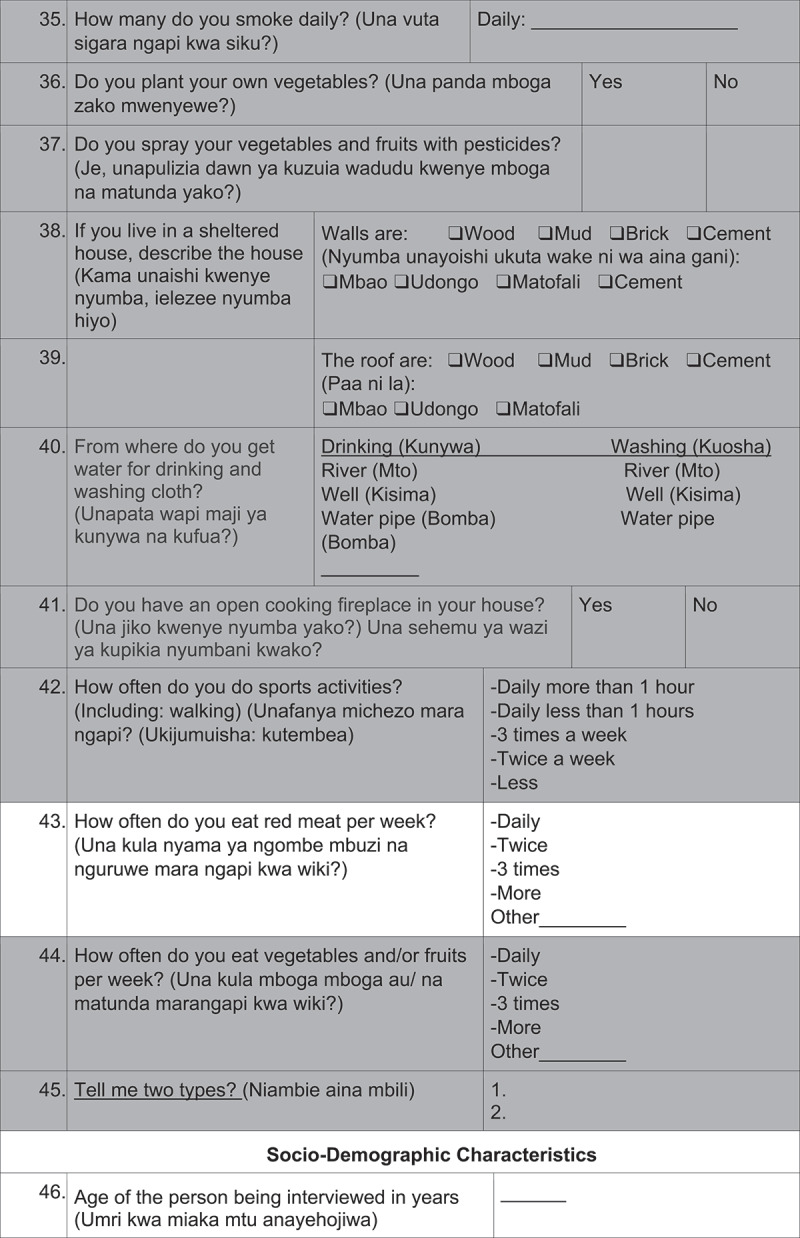
(Continued)

**Figure 2.**
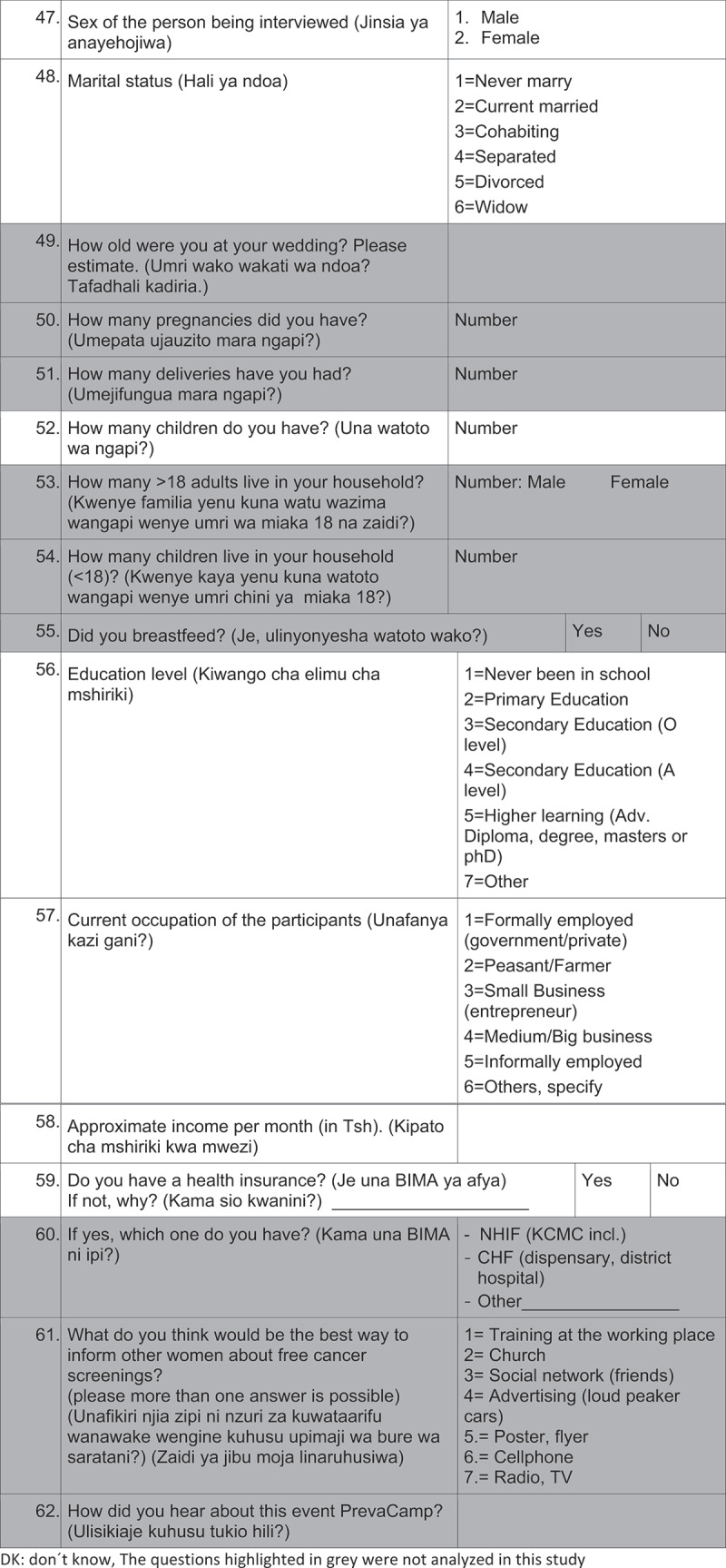
(Continued)

VIA screening: Each woman, who enrolled in the VIA screening program, was documented in a register ‘Cervical Cancer screening register’ from the MoHSW. Information recorded in a reporting form included: serial number, name of clients, address, phone number, age, first sexual contact, HIV status, date last menstrual period and screening results, use of cryotherapy and referral for Loop Electrosurgical Excision Procedure (LEEP) or other procedures. VIA screenings were performed by 6 gynecologists and specialized nurses who underwent VIA training in the past and who had longstanding experience in applying VIA screening.

### Data analysis

(Questionnaire, VIA screening): For data entry and analysis SPSS Version 23.0 was used: Continuous variables were summarized using the mean, standard deviation, median and interquartile range. Categorical data were summarized using frequency and percentage measures. The data were stratified by urban and rural areas. The comparison of the difference between the social demographic characteristics was conducted using odds ratio (OR) and 95% CIs. Chi-square was used to find possible associations between sociodemographic factors with women’s knowledge of cervical cancer, HPV, and status ‘never screened for cervical cancer’. The ‘level of knowledge of cervical cancer’ was determined by categorizing responses in to knowledgeable (at least 2 out of 4 correct answers) and nescience (less than 2 correct answers). The level of knowledge of HPV was defined as knowledgeable, if 2 out of 3 questions were correctly answered and nescience, if less than 2 questions were answered correctly.

Using the Tanzanian National Bureau of Statistics definitions, residency of participants was categorized as either ‘urban’ (Arusha and Moshi Urban) or ‘rural’ (all other sites) [26].

### Ethical considerations

Ethical research clearance was secured from Kilimanjaro Christian Medical College in Moshi, Tanzania. Participants were informed about the purpose of the questionnaires and possible outcomes of VIA screening. Arrangements were made for treatment cost coverage in the event of positive screening results that would need to undergo therapy other than cryotherapy. Consent was given prior to data collection.

## Results

The convenience sampling of all 2,807 female PrevACamps attendees included 2,192 interviewees and 2,224 screened women (Table 1). The sociodemographic characteristics of interviewed women displayed an overall mean age of 44 years (M = 44, SD = 15). 1,633 (75%) resided in rural areas. 944 (43%) were small-scale farmers, 453 (21%) were formally employed, and 531 (24.2%) had small businesses. The monthly income for 1,295 interviewees (59%) was lower than 50, USD 394 (18%) had a monthly income between 50 USD-$100, and 220 (10%) had between 100 USD-$250. 1,561 (71%) had a primary education or lower, 1,230 (56%) had no health insurance, 1,544 (70%) were married and 863 (39%) had more than 4 children.

**Table 1.** Overview about PrevACamp attendees

No	Regions	Districts	Sites	Number of attendees	Interviewees (%)	HIV infected women	VIA screened women	VIA positivity (%)	Cryo- therapy	Referral for LEEP to KCMC	Health Insurance (%)
1.	**Kilimanjaro**	Moshi urban	Moshi Urban	1081	916(42)	23	875	30(3,4)	16	14	**422(46)**
2.		Hai	Machame	252	185(8)	2	20	5(2,5)	3	2	**103(56)**
3.		Moshi rural	TPC	125	116(5)	4	125	3(2,4)	0	3	**45(39)**
4.		Siha District	Sanya Juu	232	165(8)	3	178	5(2,8)	4	1	**40(24)**
5.		Moshi rural	Mahoma	381	248(11)	10	249	11(4,4)	5	6	**67(27)**
6.		Rombo	Huruma (Rombo)	335	284(13)	3	255	7(2,7)	6	1	**154(54)**
7.		Neema (Mwanga)	Mwanga	187	120(6)	6	176	6(3,5)	0	6	**55(46)**
8.	**Arusha**	Arusha urban	Arusha urban	214	158(7)	6	164	2(1,2)	2	0	**76(48)**
	**Total**			**2,807**	**2,192(78)**	**57**	**2,224**	**69(3,1)**	**36**	**33**	**962(44)**

KCMC = Kilimanjaro Christian Medical Centre, VIA = Visual inspection using acetic acid, LEEP = Loop Electrosurgical Excision Procedure.

Differences have been seen regarding residence status and sociodemographic factors: Rural women were significantly less likely to have a secondary education (n = 1,237; 76%; p < 0.0001) and less likely to be employed (n = 1,321; 81%; p < 0.002) than urban women. Reported first sexual intercourse under 15 years was more common in urban areas (n = 23; 4.2%) than in rural areas (n = 60; 3.8%). 962 (44%) women had health insurance, with no remarkable difference between urban and rural regions (Table 1).

### Cancer knowledge/misconceptions

The level of overall cancer knowledge is shown in Table 2. 1,785 (81%) of 2,192 women had heard about cancer with a noticeable difference between urban and rural areas. 1,151 (53%) of the interviewees reported that cancer is a problem in their community. 598 (27%) had a family history of cancer deaths (Table 2).

**Table 2.** Behavioral risk factors, women’s knowledge about cancer, cancer risk factors, cervical cancer and HPV by residence

Variables	Total N (%)	Urban N (%)	Rural N (%)	OR (95%CI)	p-value
**Behavioral Risk Factors**					
Cigarette Smoking	Total	Urban	Rural		
No	**2,136(97.4)**	543(97.1)	1,593(97.6		
Yes	**56(2.6)**	16(2.9)	40(2.4)	1.17(0.65–2.11)	0.593
Alcohol use					
No	**1,174(53.6)**	300(97.1)	874(97.6)		
Yes	**1,018(46.4)**	259(2.9)	759(2.4	0.99(0.82–1.21)	0.952
Red meat intake weekly					
Less/none	**1,742(79.5)**	422(75.5)	1,320(80.8)		
More/daily	**450(20.5)**	137(24.5)	313(19.2)	**1.39(1.09–1.72)**	**<0.0070**
**Knowledge about cancer**					
Household members with cancer currently	Total	Urban	Rural		
No	**1887(86.1)**	488(87.3)	1,399(85.7)		
Yes	**305(13.9)**	71(12.7)	234(14.3)	0.87(0.65–1.16)	0.337
History of household members with cancer					
No	**1,594(72.7)**	380(68.0)	1,214(74.3)		
Yes	**598(27.3**	179(32.0)	419(25.7)	**1.36(1.11–1.68)**	**<0.004**
Cancer problem in the community					
No	**1,041(47.5)**	275(49.2)	766(46.9)		
Yes	**1,151(52.5)**	284(50.8)	867(53.1)	0.91(075–1.11)	0.350
Heard about cancer before					
No	**407(18.6)**	74(13.2)	333(20.4)		
Yes	**1,785(81.4)**	485(86.8)	1,300(79.6)	**1.68(1.28–2.21)**	**<0.0002**
Misconceptions in getting cancer	Multiple answers	N = 164	N = 781		
Curse	**115(12.3)**	10(6.1)	105(13.6)	**2.39(1.22–4.70)**	**<0.0089**
Genetic mutation	**403(43.1)**	88(53.7)	315(40.9)	**0.58(0.42–0.82)**	**<0.0017**
Direct contact	26**(2.8)**	5(3.0)	21(2.7)	0.88(0.33–2.37)	0.7979
Vaccine	**54(5.8)**	2(1.2)	52(6.7)	**5.78(1.38–24.12)**	**<0.0064**
Contraceptive use	**288(20.4)**	37(22.6)	251(32.6)	**1.63(1.09–2.42)**	**<0.0155**
**Knowledge about cervical cancer**					
Knowledge about CC	Total	Urban	Rural		
Poor	**1,708(77.9)**	430(76.9)	1,278(78.3)		
Good	**484(22.1)**	129(23.1)	355(21.7)	1.08(0.86–1.36)	0.510
Heard about CC prior PrevACamp					
No	**731(33.3)**	178(31.8)	553(33.9)		
Yes	**1,461(66.7)**	381(68.2)	1,080(66.1)	1.07(0.87–1.32)	0.5093
Screened for CC prior PrevACamp					
No	**1,942(88.6)**	468(83.7)	1,474(90.3)		
Yes	**250(11.4)**	91(16.3)	159(9.7)	**1.80(1.36–2.38)**	**<0.0001**
Risk factors for CC					
Not aware	**2009(91.7)**	521(93.2)	1,488(91.1)		
Aware	**183(8.3)**	38(6.8)	145(8.9)	**0.75(0.52–1.08)**	**<0.1247**
Lower risk for CC					
Not aware	**2057(93.8)**	526(94.1)	1,531(93.8)		
Aware	**135(6.2)**	33(5.9)	102(6.2)	**0.94(0.63–1.41)**	**<0.7711**
Symptoms about CC					
Not aware	**1,671(76.2)**	424(75.8)	1,247(76.4)	ref	
Aware	**521(23.8)**	135(24.2)	386(23.6)	1.03(0.82–1.29)	0.8058
Vaginal bleeding after menopause is a sign of CC					
Not aware	**1174(53.6)**	299(53.5)	875(53.6)	ref	
Aware	**1,018(46.4)**	260(46.5)	758(46.4)	1.01(0.83–1.22)	0.9693
**Knowledge of Human Papillomavirus**					
Heard about HPV infection	Total	Urban	Rural		
Not aware	**1,693(77.2)**	433(77.5)	1,26,077.2)		
Aware	**499(22.8)**	126(22.5)	373(22.8)	0.98(0.78–1.24)	0.8835
Heard about HPV vaccines					
Not aware	**1,175(53.6)**	316(56.5)	859(52.6)		
Aware	**1,017(46.4)**	243(43.5)	774(47.4)	0.85(0.70–1.04)	01082
Knowledge about HPV					
Poor	**1,548(70.6)**	414(74.1)	1,134(69.4)		
Good	**644(29.4)**	145(25.9)	499(30.6)	**0.8.(0.64–0.99)**	**<0.039**

CC = Cervical Cancer, HPV = Human Papillomavirus, PrevACamp = Cancer and Awareness Campaign.

Out of 5 multiple choice questions about beliefs and misbeliefs about the etiology of cancer: 403 (43%) responded ‘genetic mutation’ as a cause of cancer, followed by use of contraceptives 288 (20%), curse 105 (14%), 54 (5.8%) vaccine, and direct contact with a cancer patient 26 (2.8%). Incorrect responses were positively associated with women living in rural settings (Table 2).

### Knowledge of cervical cancer

**Figure 3.**
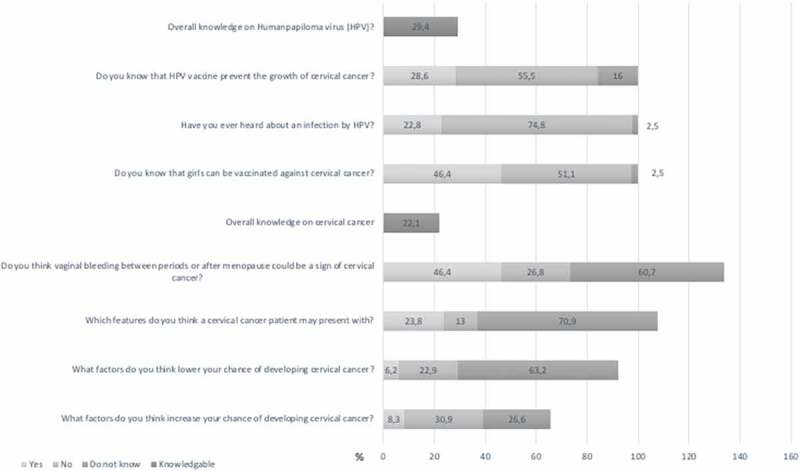
Knowledge level of cervical cancer and HPV

**Figure 4.**
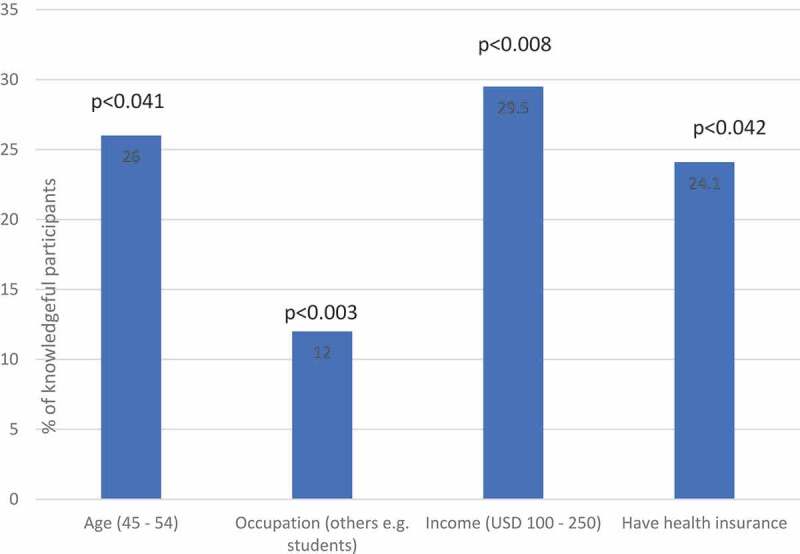
Association between social demographic characteristics and Cervical Cancer knowledge

The level of knowledge about cervical cancer was as follows: Among 2,192 women interviewed, 731(33%) reported that they had never heard of cervical cancer. 484 (22%) were knowledgeable (Figure 3). The following factors had a significant impact on cervical cancer knowledge: Women aged between 45–54 years, had a monthly income between 100 USD and 250 USD, and had health insurance (Figure 4). Education level, rural or urban residence, and the number of children were not associated with better knowledge of cervical cancer (Table 3). 16.3% women living in urban and 9.7% of women living in rural areas had been screened for cervical cancer prior to PrevACamp (Table 2).

**Table 3.** Association between sociodemographic characteristics and cervical cancer/HPV knowledge

	Total	Knowledge of CC			Knowledge of HPV		
Variables	N	N(%)	OR(95%CI)	p-value	N(%)	OR(95%CI)	p-value
**Age, years**							
<25	194	36(18.6)	1.00		52(26.8)	1.00	
25–34	460	110(23.9)	1.38(0.91–2.10)	0.134	152(33.0)	1.35(0.93–1.96)	0.116
35–44	571	122(21.4)	1.19(0.79–1.80)	0.404	181(31.7)	1.27(0.88–1.82)	0.201
45–54	469	122(26.0)	**1.54(1.02–2.34)**	**<0.041**	151(32.2)	1.30(0.89–1.88)	0.171
55+	498	94(18.9)	1.02(0.67–1.56)	0.923	108(21.7)	0.76(0.52–1.11)	0.152
**Education level**							
Never been in school	131	28(21.4)	1.00		19(14.5)	1.00	
Primary education	1430	289(20.2)	0.93(0.60–1.44)	0.751	382(26.7)	**2.15(1.30–3.54)**	**<0.003**
Secondary and above	631	167(26.5)	1.32(0.84–2.08)	0.225	243(38.5)	**3.69(2.21–6.16)**	**<0.0001**
**Occupation**							
Peasant/farmer	944	207(21.9)	1.00		261(27.6)	1.00	
Business	542	120(22.1)	1.01(0.78)	0.924	156(28.8)	1.06(0.83–1.34)	0.640
Employed	523	135(25.8)	1.24(0.97–1.59)	0.092	182(34.8)	**1.40(1.11–1.76)**	**<0.004**
Other e.g. students	183	22(12.0)	**0.49(0.30–0.78)**	**<0.003**	45(24.6)	0.85(0.59–1.23)	0.395
**Level of income (USD $)**							
<50	1295	277(21.4)	1.00		353(27.3)	1.00	
50-<100	394	76(19.3)	0.88(0.66–1.17)	0.369	102(25.9)	0.93(0.72–1.20)	0.591
100–250	220	65(29.5)	**1.54(1.12–2.12)**	**<0.008**	85(38.6)	**1.68(1.25–2.26)**	**<0.001**
>250	109	32(29.4)	1.53(0.99–2.36)	0.055	45(41.3)	**1.88(1.26–2.80)**	**<0.002**
Unknown	174	34(19.5)	0.89(0.60–1.33)	0.575	59(33.9)	1.37(0.98–1.92)	0.068
**Health Insurance**							
Yes	962	232(24.1))	**1.23(1.01–1.51)**	**<0.042**	310(32.2)	**1.28(1.06–1.53)**	**<0.010**
No	1230	252(20.5)	1.00		334(27.2)	1.00	
**Residence**							
Urban	559	129(23.1)	1.08(0.86–1.36)	0.510	145(25.9)	0.80(0.64–0.99)	**<0.039**
Rural	1633	355(21.7)	1.00		499(30.6)	1.00	
**Children**							
No	215	39(18.1)	1.00		68(31.6)	1.00	
Yes	1977	445(22.5)	1.31(0.91–1.88)	0.4143	576(29.1)	0.89(0.66–1.20)	0.446
**Ever screened for CC**							
No	1942	399(20.5)	1.00		546(28.1)	1.00	
Yes	250	85(34.0)	**1.99(1.50–2.65)**	**<0.0001**	98(39.2)	**1.65(1.26–2.16)**	**<0.0001**

CC = Cervical Cancer, HPV = Human Papillomavirus.

### Knowledge on HPV

**Figure 5.**
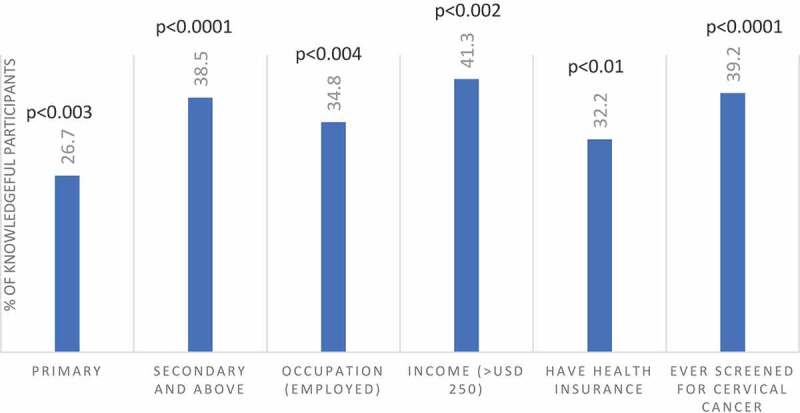
Association between social characteristics and HPV knowledge

635 (29%) were knowledgeable about HPV. 1,644 (75%) had never heard about HPV infection. 1,118 (51%) did not know that girls can be vaccinated against cervical cancer (Figure 2). The following factors were found to have a significant impact on knowledge of HPV: Women with primary education and above, being employed, had income between 100 USD and 250 USD/month, had health insurance, and screened for cervical cancer before (Figure 5). The number of children had no association with HPV knowledge (Table 3). Women living in urban areas had less knowledge about HPV compared to rural women (Table 2).

### VIA screening outcome

2,246 (80%) from 2,807 female PrevACamps attendees enrolled voluntarily for VIA-screening. 26 women were excluded from VIA because they were under 18 years of age (22), pregnancy (1), menstruation (2) and history of TAH (1). A total of 2,224 women were screened (urban: 1,039, rural: 1,185). The number of HIV infected women was 57 (2.6%). The overall mean age group was between 35 and 44 years. Among these, the proportion of positive VIA was 69 (3.1%) (Urban: 32, rural: 37, p-value = >0.478). 36 underwent cryotherapy, and 33 were referred to KCMC for LEEP or further investigations/diagnosis (Figure 1).

## Discussion

This study accompanied PrevACamp in its real-world setting with the aim to gain a deeper understanding of cervical cancer and HPV knowledge among women attending a voluntarily screening program for future adaptations of preventive measures. The second goal of this study was to determine the prevalence of pre-cancerous cervical lesions among the attendees to identify possible regions or high-risk populations.

Our finding highlight (1) nescience on cervical cancer regardless of education level, resident status and the number of children, (2) nescience on HPV in all age groups and especially in urban areas (3) and misconception about cancer.

### Cervical cancer and HPV knowledge

A third of the interviewees had never heard about cervical cancer. This compares to previous studies in Tanzania, such as a Kilimanjaro-region-based study (in semi-rural and urban areas), a study from Lake Zone and a representative country-wide survey. These studies found only 17%, 16.9% and 15% of women respectively had never heard of cervical cancer disease [7,22,27]. Our findings also show no significant difference in cervical cancer knowledge between women living in urban or rural areas as has been found in other studies [14,21,22,28]. In addition, our results demonstrate that only 22% and 29% of women showed knowledge about cervical cancer and HPV, respectively. These findings are in line with studies from other SSA settings [7,18,22,27,29–31].

Surprisingly, women living in urban areas had less knowledge about HPV compared to rural women (Table 2), which might be carefully interpreted by the presence of many faith-based hospitals, NGO´s and other health facilities [32] in rural Kilimanjaro, which is unique for Tanzanian rural areas.

Apart from the afore mentioned, we found significant differences in the level of cervical cancer knowledge in our study population. Women with higher income, health insurance, and previous experience of VIA screening had significantly more knowledge about cervical cancer. This is possible because women who have health insurance may have better health-seeking behavior and have had more interaction with health facilities. These findings are in tandem with results from a study from Zanzibar [30]. Another study from Zimbabwe found that women with a higher income, and who had more contact with the health care system had better cervical cancer knowledge [29].

Education level and employment appear to play no role in the knowledge about cervical cancer in our study. These results are not consistent with other studies in SSA [7,22,30,31]. Also, no correlation was found between multiparous and cervical cancer knowledge, as has been reported in previous studies from Tanzania [22,32]. However, a study from India documented that a high number of pregnancies, using family planning, and frequent contact with the health-care system found to be associated with increased access of cervical cancer screening services [33]. A possible explanation for the differences in our study with previous studies could be that our study population was not a representative sample or a hospital patient population, which can be assumed to have higher health-seeking behavior. Following this line of reasoning, the PrevACamps reached people with less than average knowledge and hence the desired target group for an intervention program was addressed.

A second explanation could be the timing of the previously conducted studies: the NCCS by MoHSW was introduced in 2013 [15,22]. In the years before and after NCCS implementation (mainly in the years of 2012 to 2015), cervical cancer and screening programs received great nationwide attention through mass media [22] and increased governmental support, especially from the former First Lady Her Excellency Salma Kikwete [34,35].

Following the logic of influences in mass media and government engagement, we consequently see higher knowledge regarding HPV vaccination compared to Cervical Cancer knowledge in our study population. During the PrevACamps, HPV vaccination implementation campaigns in schools were conducted country-wide [16,17], and announcements through mass media were frequently given.

### Misconception about cancer

Another major barrier to combat cancer is the misconception about cancer. Our study found that every second woman living in rural areas has misconceptions about cancer which may lead to disbelief and heedlessness toward cancer prevention [19,36]. The influence of sociocultural beliefs in relation to cervical cancer misconceptions has been studied before but with inconsistent findings. McCree et al. found key stakeholders in Tanzania believed that the perception of low resources was a stronger barrier than the impact of folk myths and sociocultural-based misconceptions [34]. This is contradicted with Zambia’s report where folk myths and misconceptions lead to poor utilization in cancer education and screening services [37]. Cervical cancer may impact HIV infected women differently than other populations. Studies show that women infected with HIV are more likely to appear with cervical cancer disease later in life [38,39]. Bateman et al. assessed barriers to cervical cancer screening among HIV infected women in Tanzania and found that women had high misconceptions of cervical cancer screening and felt that diagnosis may lead to death, hence hindering women from seeking health care [39].

### Need for extended cancer education and screening programs

PrevACamp was the first community-based prevention and awareness cancer campaign organized by CCC in Northern Tanzania. The framework combined cancer education seminars and screenings for women, especially in remote areas.

Considering 75% of reproductive-aged women live in rural areas [28], outreach programs covering these areas are crucial [21,40], especially as higher rates of family history of cancer were reported in rural areas. Our study found that 76% of women residing in rural areas were not able to identify any early symptoms, risks, or preventive factors of cancer. Apart from this, previous studies have shown that access to health care differs between women in rural and urban areas, largely due to transportation and financial constraints that prevent screening attendance [21,40,41]. Therefore, enhancement of cancer knowledge, that is needed to increase women’s health, might be best achieved by bringing screening and education into the rural areas.

Comparatively, in a review from Runge et al. with a cumulative VIA positivity rate of 9.2%, our VIA positivity rate was 3.1% [14]. However, the reviewed studies showed vast difference with VIA positive screening results ranging from 4.3% (with the study setting in Dar es Salaam, in Dar es Salaam/Pwani and Mwanza/Mtwara) to 12.9% (in Mwanza/Mara). Just like prior PrevACamps, the VIA screening programs targeted the general population and were announced in public [14]. The differences with low VIA positive screening results could be explained by the high density in primary health care facilities including HIV clinics in these settings [32]. This might also influence the lower VIA positivity in our study with only 2.6% of HIV infected participants, compared with the study from Mara where 8.2% were infected with HIV [38]. Furthermore, PrevACamp findings showed no significant difference between residence status and VIA-positivity. Following this line, the setting in our study has the highest density of primary health care facilities after Dar es Salaam and also a long-standing tradition of faith-based hospitals [42]. The health system coverage in our setting may as well be a positive impact on HIV patients and influence the lower VIA positivity rate.

## Limitations

Study findings cannot be generalized for the Tanzanian population as our sample represents voluntarily attending women from Northern Tanzania. During VIA screening, women above the age of 18 years were enrolled. However, WHO guidelines recommend screening at age 30 years old onwards. This might have also contributed to a lower positive VIA screening outcome.

## Conclusion

Our findings show a lack of cervical cancer and HPV knowledge among women in two regions in northern Tanzania. This poor knowledge is alarming and requires collaborative efforts from different stakeholders including health care providers, policymakers, and non-governmental organizations to increase cancer knowledge within the communities. Education-based cancer knowledge programs and mass screening programs, especially in remote areas should be considered, as this approach will reach the underserved rural population. Future cancer programs that strengthen the collaboration with public schools for primary and secondary prevention and to extend special cancer education programs on mass media and loudspeaker cars should also be considered.

Educational program to raise knowledge about HIV infections in the community is also needed to reach a standard level of knowledge and understanding about the importance of HIV prevention, treatment and cervical cancer screening. Another step would be to set up more cervical cancer screening centers in the primary health care in remote areas, collaboration of multiple stakeholders such as cancer survivors and community health care workers are essential with sufficient screening equipment’s. Provided, cancer care staff workloads do not increase, added funds for health care providers for cancer awareness training are required [17].

## Paper context

14.9 million women in Tanzania are at risk of developing cervical cancer, the most prevalent cancer among women in this East African country. Late-stage presentation contributes to high mortality rates. Between 2017 and 2019, a study was conducted amongst 2,807 female participants of prevention and awareness campaigns in northern Tanzania. Results display significant nescience on cervical cancer and HPV. Outreach programs, especially in rural areas, seem to target the population in need of health education.
